# Rescue upon rescue: ventricular tachycardia ablation using cerebral protection device in a patient with left ventricular thrombus associated with mitral valve transcatheter repair

**DOI:** 10.1007/s10840-025-02192-8

**Published:** 2025-11-25

**Authors:** Elie Kozaily, Bishnu P. Dhakal, Ian Laxina, Rory P. Dowd, Chau Vo, Jeffrey Winterfield

**Affiliations:** Hank and Laurel Greer Chair in Cardiac Electrophysiology Chief, Cardiac Rhythm Services, Medical University of South Carolina (MUSC), Charleston, SC USA

**Keywords:** Ventricular tachycardia, Heart failure, Ventricular tachycardia ablation

## Abstract

**Abstract:**

We present the case of an elderly man with advanced heart failure and worsening severe mitral regurgitation who underwent transcatheter mitral valve edge-to-edge repair (M-TEER) who presented with VT storm 2 weeks after M-TEER. VT ablation was considered but not performed since cardiac CT scan revealed M-TEER associated thrombus on the ventricular surface of the posterior mitral leaflet. Despite being on amiodarone, the patient returned few days later with VT storm. After multidisciplinary assessment and patient-centric discussions, the decision was made to proceed with emergent, rescue VT ablation using cerebral embolic protection device (CEP) and mechanical support as needed. VT ablation was safe and successfully performed. Patient had no recurrence of VT during 2.5 month follow up. To our knowledge, this is the first reported case of VT ablation using CEP for LV thrombus in North America.

**Capsule abstract:**

Emergent, successful VT ablation in advanced HF patient with transcatheter mitral prosthesis associated left ventricular thrombus using cerebral protection device.

We present the case of a 78-year-old man with metabolic syndrome associated chronic kidney disease KDIGO stage 3b, ischemic cardiomyopathy with two remote coronary artery bypass graft surgeries (left internal mammary artery to left anterior descending (LAD) artery, and saphenous venous graft (SVG) to LAD and SVG to diagonal coronary artery), advanced stage heart failure with reduced ejection fraction (HFrEF) ACC/AHA stage D, ventricular tachycardia (VT) on chronic amiodarone, atrioventricular block requiring biventricular pacing, and moderate to severe mitral regurgitation. He had lack of improvement in left ventricular (LV) function despite guideline directed medical therapy (GDMT). He has severe underlying native coronary disease not amenable to percutaneous revascularization with occluded LIMA to LAD.

His first episode of VT happened 6 years ago, leading up to upgrade from pacemaker to biventricular cardiac defibrillator for secondary prevention. He has required chronic amiodarone therapy for episodes of VT. One month prior to VT storm presentation, given persistent HF symptoms with maximally tolerated GDMT, the patient underwent transcatheter mitral valve edge-to-edge repair (M-TEER) for severe secondary mitral regurgitation (MR). During the procedure, the patient developed VT at 120 bpm which was terminated with anti-tachycardia pacing from his device.

Four weeks following M-TEER, the patient presented with multiple symptomatic VT episodes despite being on amiodarone. InHeart cardiac CT scan (Bordeaux, France) and echocardiography revealed 1.8 cm thrombus on the ventricular side of the posterior mitral valve prosthesis with mild eccentric MR. LV inferior and inferolateral walls were akinetic with evidence of transmural delayed hyperenhancement on CT scan. Antiarrhythmic drug (AAD) therapy was titrated, with addition of mexiletine. VT ablation was considered but differed given associated risks of embolization with LV thrombus. The patient was deemed not a candidate for advanced cardiac therapies given advanced age, prior sternotomies and poor renal function.

His ventricular arrhythmia worsened despite escalation in antiarrhythmic medication (Figs. 1A and 2). He was then started on IV lidocaine and amiodarone and multidisciplinary team including advanced heart failure, interventional and structural cardiologists and electrophysiologist was convened. His PAAINESD risk score was elevated to 26 indicating a high risk of hemodynamic compromise peri-procedurally. After extensive discussion with the patient and his family, the decision was made to proceed with rescue, emergent VT ablation with cerebral embolic protection (CEP) device and mechanical circulatory support if needed.

**Fig. 1:**
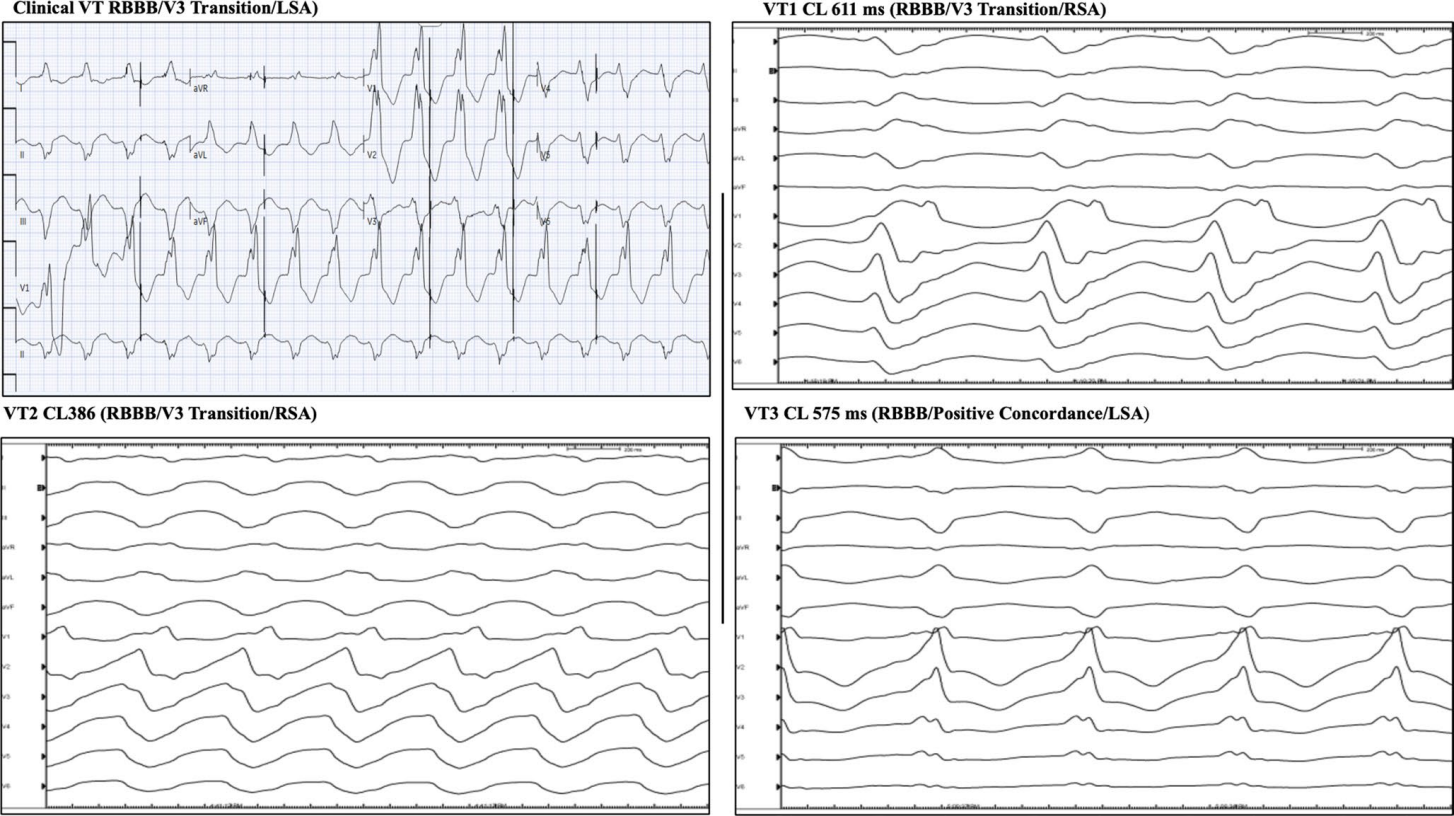
Clinical VT with VT morphologies during VT ablation

**Fig. 2:**
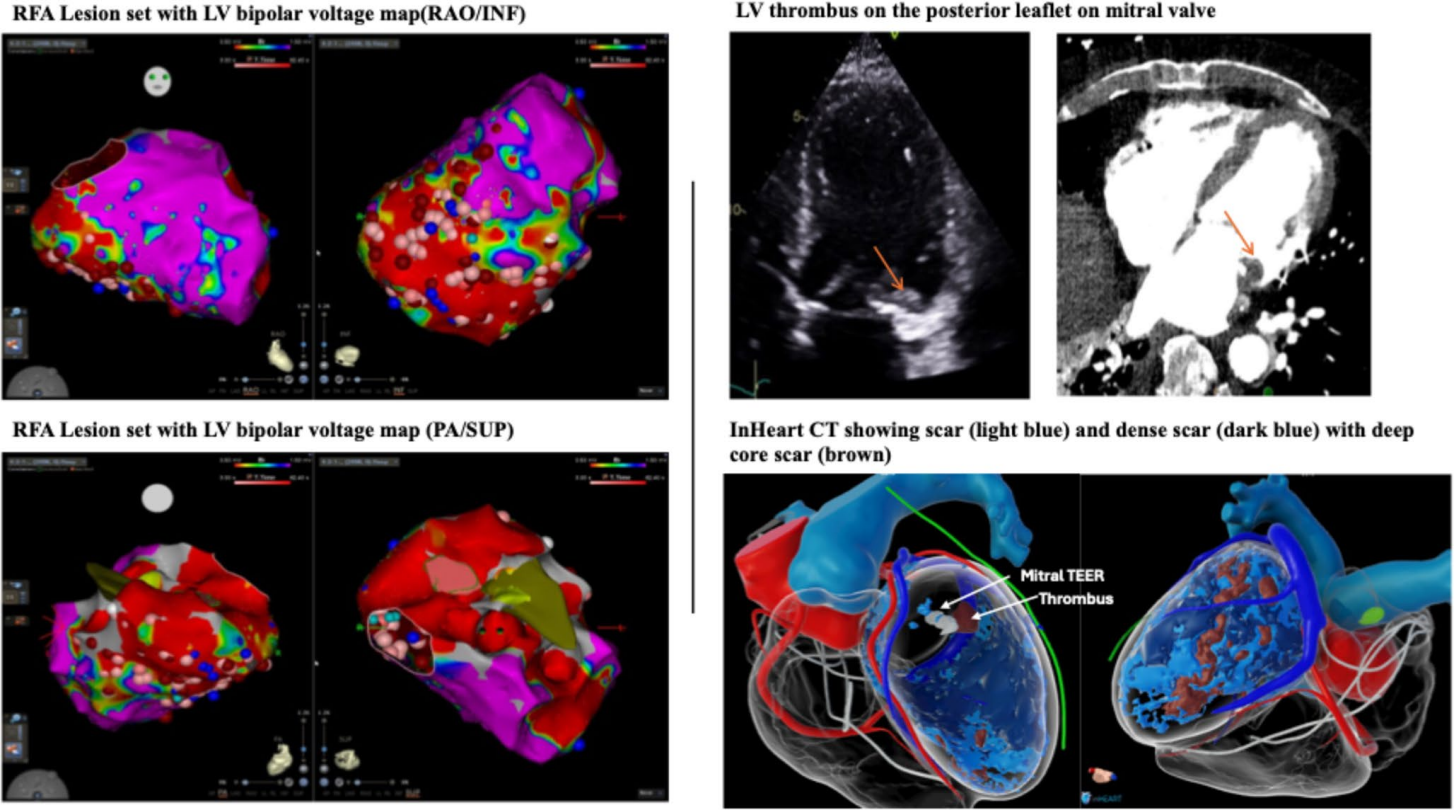
Electro-anatomical Mapping during VT ablation with echocardiogram and InHeart CT

Within 48 h of presentation, the patient was taken to the EP lab. After general anesthesia administration, CEP Sentinel (Boston Scientific) was inserted via right radial arterial access. A 90-minute timer was then started to remain compliant with time limits of Sentinel device. Patient was inducible for 3 different VT spontaneously (Fig. 1). ICE imaging demonstrated large inferior LV scar extending from base to apex, necrotic posteromedial papillary muscle and anterolateral papillary muscle with the M-TEER clip. The decision was made to access the LV in a retrograde fashion given a recent transseptal approach for M-TEER, especially with associated thrombus. Catheter placement in the LV easily induced VT 1. VT1 spontaneously transitioned to VT2 and VT3. Entrainment and limited activation mapping was performed due to switching of VTs. The entrance to VT1 was noted on the basal lateral inferior scar with exit on the base of the posteromedial papillary muscle. Radiofrequency ablation (RFA) energy was delivered to the mid inferior LV with the Thermoccol STSF catheter (Biosense Webster) in power control mode with power titrated from 30 to 40 W with careful monitoring of impedance change and contact with ICE and force sensor. Lesions in this area resulted in slowing and termination of VT1. Subsequently, pace map matches for VT1-VT3 with RF energy were targeted.

We remapped the LV in RV paced rhythm and collected simultaneous FAM, Voltage and ILAM mapping data (Fig. 2). Additional areas of late potentials, local abnormal ventricular activity, and pacemapped sites in the inferior wall were targeted in the inferior wall. With further programmed electrical stimulation, a faster VT4 was induced and ultimately cardioverted at 360 Joules. No further mapping or ablation was pursued given time limitations with Sentinel device.

Following ablation and withdrawal of catheters from the LV, the patient remained electrically stable. There was no pericardial effusion on ICE pre or post ablation. The Sentinel device was removed by the interventional team without evidence of embolic thrombus. Transthoracic echocardiogram re-demonstrated sub-mitral thrombus, TEER device in appropriate place and unchanged mild mitral regurgitation. Due to signs of cardiogenic shock with pressor requirement at end of case, the interventional team placed an intra-aortic balloon pump (IABP) through the right femoral arterial access site.

The patient was allowed to awake from anesthesia and returned to the cardiac ICU. After 48 h of close clinical neurologic and hemodynamic assessment, the IABP was weaned and removed. The patient was discharged after optimization of HF medical therapy and home health services were arranged. His discharge medication included amiodarone 200 mg once daily, beta-blockers and oral anticoagulants.

Patient was closely followed up in heart failure and EP clinic and he is doing well with no recurrent VT episodes during 2.5 months after the procedure.

## Discussion

VT ablation in the setting of left sided thrombus LV and/or left atrial appendage (LAA) was reported in a limited number of case series. With laminated, or non-mobile intracardiac thrombi reported cases of VT ablation were overall safe without embolic complications. The use of ICE proved beneficial to mitigate risks in such situations [1–3]. Yet, in a large registry study, the rates of in hospital complications, especially cerebrovascular complications, were found to be elevated [4]. In non-urgent situations, the consensus is to initiate anticoagulation and repeat imaging before proceeding with VT ablation [5]. Nevertheless, the use of CEP devices in EP procedures including VT ablation was recently reported by Mazzone and colleagues [6]. They used 2 types of CEP (a capture device from radial artery, a deflection device from femoral artery). All 12 patients who underwent VT ablation in their series had CEP, half of them had LV thrombus while the remainder had LAA or aortic arch thrombus. The outcomes are not separated by CEP type or intracardiac thrombus location, but there were no periprocedural strokes and CEP-related complications at the arterial access site were minor and noted in 6% of the total cohort (*N* = 65).

Our patient presented with electrical storm, refractory to two antiarrhythmic medications and no advanced cardiac therapies option. Despite older age, the patient was overall functional and proceeding with VT ablation was deemed a rescue procedure. To our knowledge, this is the first reported case of VT ablation using CEP for LV thrombus in North America, especially LV thrombus that is localized on mitral valve leaflet status post M-TEER. While the procedure was high risk, pre-operative planning and peri-procedural imaging led to a safe and successful VT ablation which improved our patient’s quality of life. Collaboration within a multidisciplinary team in a referral center remains essential for such success.

## Data Availability

No datasets were generated or analysed during the current study.
